# Supplementary material for: Defocused Image Changes Signaling of Ganglion Cells in the Mouse Retina
Source: Cells. 2019 Jun 26;8(7):640. doi: 10.3390/cells8070640 (PMC6678497; doi:10.3390/cells8070640)
Supplement: Supplementary file 1 [file cells-08-00640-s001.pdf]

Supplementary Figure:

Supplementary Figure 1.

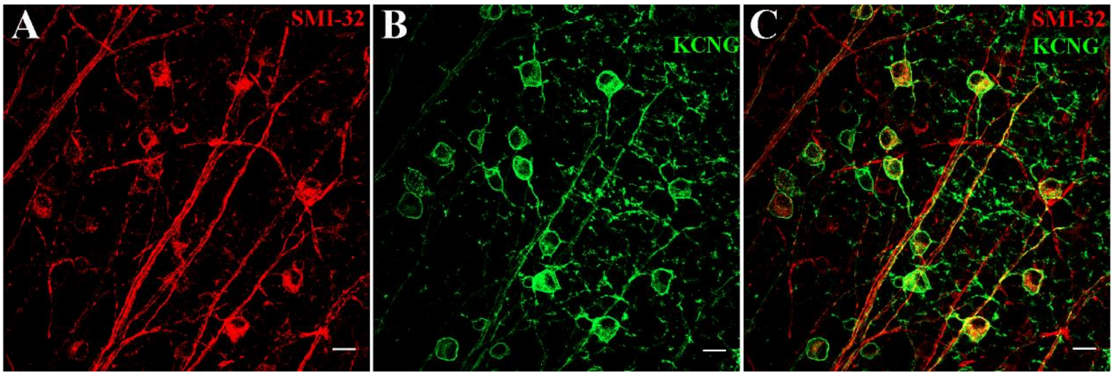

A: antibodies to neurofilaments (SMI-32) labeled α-RGCs.

B: showed KCNG4-Cre transgenic mouse with GFP labeling RGCs.

C: Merged image show KCNG4-Cre mouse labled α-RGCs.

Scale bar= 20 μm

Supplementary Figure 2. Setup for patterned light stimulation recording

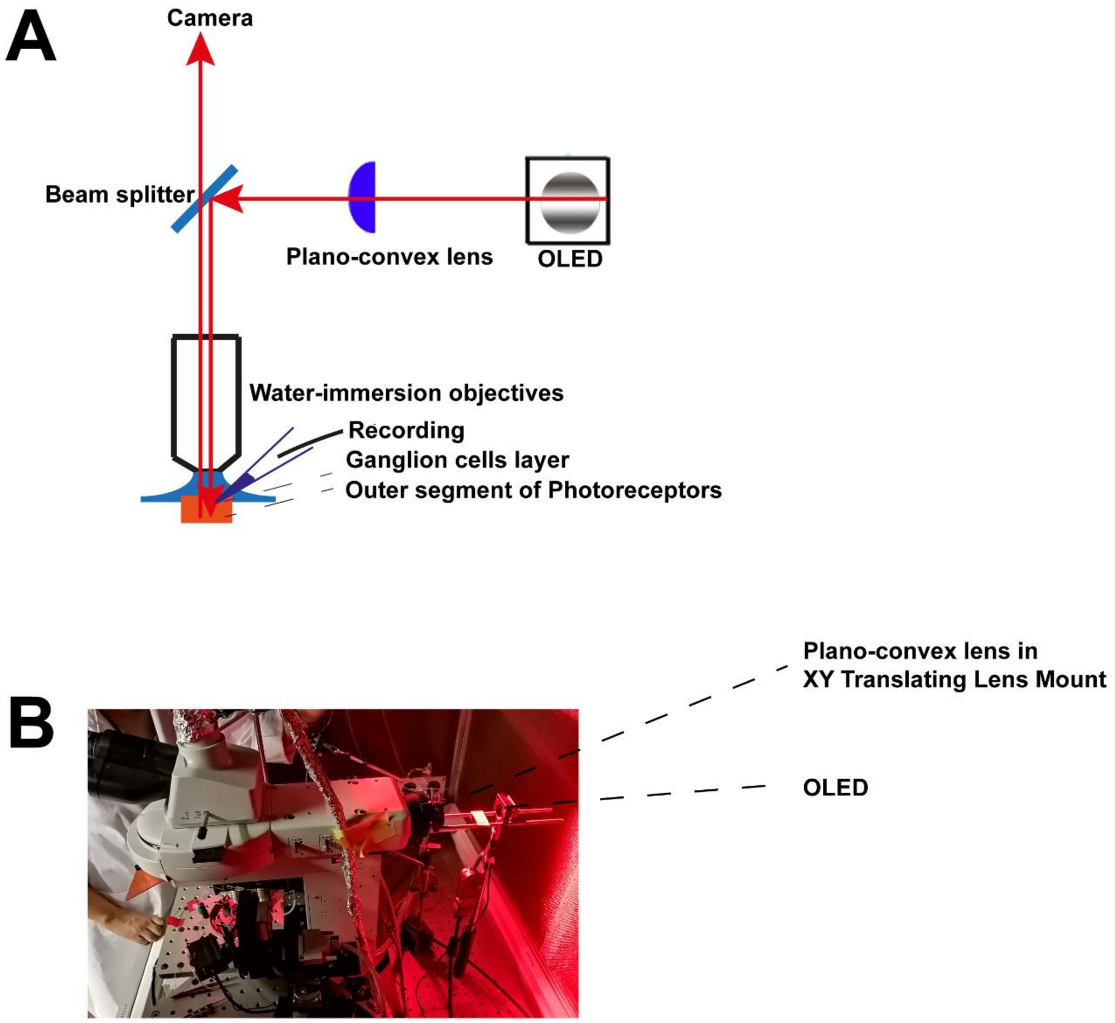

Supplementary Figure 2. Setup for patterned light stimulation recording
A: Schematic of patterned light stimulation in the configuration for ganglion cell recording.
Once the desired recording configuration has been obtained. Patterned visual stimuli from an OLED are presented to the different layer of retina through a plano-convex lens, a beam splitter, and the 40 water immersion objective.

The OLED and a CRT display are connected to the same PC and displayed in duplicate mode (both are 800×600-pixel resolution).

B: The visual stimulation arm. The OLED and the plano-convex lens are mounted via the back of epifluorescence port of the microscope.

TTL pulses are generated by the visual stimulation computer at the onset of

light stimulation, and sent via the parallel port to the Analog-to-Digital converter on the digitizer to trigger synchronized visual stimulation and electrophysiological recording.
